# Unravelling diagnostic challenges: A case report of pulmonary langerhans cell histiocytosis with atypical radiologic presentation

**DOI:** 10.1002/rcr2.70004

**Published:** 2024-09-10

**Authors:** Joseph Inauen, Simone Barry

**Affiliations:** ^1^ Department of Thoracic Medicine Royal Adelaide Hospital Adelaide South Australia Australia

**Keywords:** Langerhans cell Histiocytosis, pulmonary Langerhans cell Histiocytosis, pulmonary nodule, surgical lung biopsy

## Abstract

This clinical case highlights the diagnostic challenges encountered in a young adult smoker presenting with undifferentiated pulmonary nodules. Initial investigations were inconclusive, necessitating surgical lung biopsy due to the nodules' size and location. The histopathological examination revealed pulmonary Langerhans cell histiocytosis (PLCH). This emphasizes the importance of considering PLCH in the differential diagnosis of pulmonary nodules, particularly in smokers. Moreover, it underscores the value of surgical biopsy in cases where other diagnostic techniques are limited. Early recognition and accurate diagnosis are crucial for optimal management and outcomes in PLCH.

## Graphical abstract

This clinical case report describes the diagnostic journey of a young adult smoker presenting with undifferentiated pulmonary nodules. Despite initial challenges in diagnosis due to the nodules' size and location, surgical lung biopsy revealed pulmonary Langerhans cell histiocytosis (PLCH). This highlights the importance of considering PLCH in the differential diagnosis of pulmonary nodules and underscores the value of surgical biopsy in challenging cases.

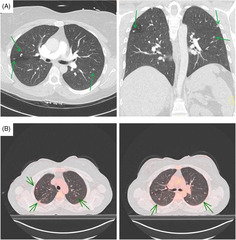

## INTRODUCTION

Pulmonary Langerhans cell histiocytosis (PLCH) is a rare form of interstitial lung disease with an estimated prevalence of 1–2 per million.^1^ It shares histologic features with other forms of Langerhans cell histiocytosis (LCH) which can affect multiple organs and remains largely of unknown aetiology.^2^ Unlike LCH, which is primarily seen in children, PLCH predominantly occurs in young adults, particularly in those who currently smoke, and is largely confined to the lungs. This condition is characterized by inflammatory lesions that surround bronchioles and cause destruction of bronchiolar wall and adjacent lung tissue,^3^ seen radiologically as cysts, nodules, and cavitary lesions. Langerhans cells are antigen presenting cells found predominantly in skin and mucosa. In LCH, they are found in distinct granulomas that may, in some cases, represent malignant monoclonal proliferation.^2^, ^4^

Here we present a case of PLCH that showed a unique radiologic nodular pattern, posing diagnostic challenges, advocating for a diagnostic surgical biopsy.

## CASE REPORT

A 37‐year‐old female was referred to the respiratory clinic for investigation of multiple lung nodules found on computed tomography (CT) pulmonary angiogram that was undertaken in a rural town to investigate chest pain. She reported a cough with occasional sputum production, although no dyspnoea. Concerningly, she reported unintentional weight loss of up to 20 Km over the preceding 5 months.

Her medical history revealed a previous Pap smear that showed low‐grade cervical dysplasia (CIN1) although subsequent screenings were negative. A recent colonoscopy was unremarkable in the setting of a family history of Crohn's disease. There was no other significant medical history nor family history. At the time of presentation, she was an active cigarette smoker, smoking up to 40 cigarettes per day. She had no relevant occupational exposures.

**FIGURE 1:**
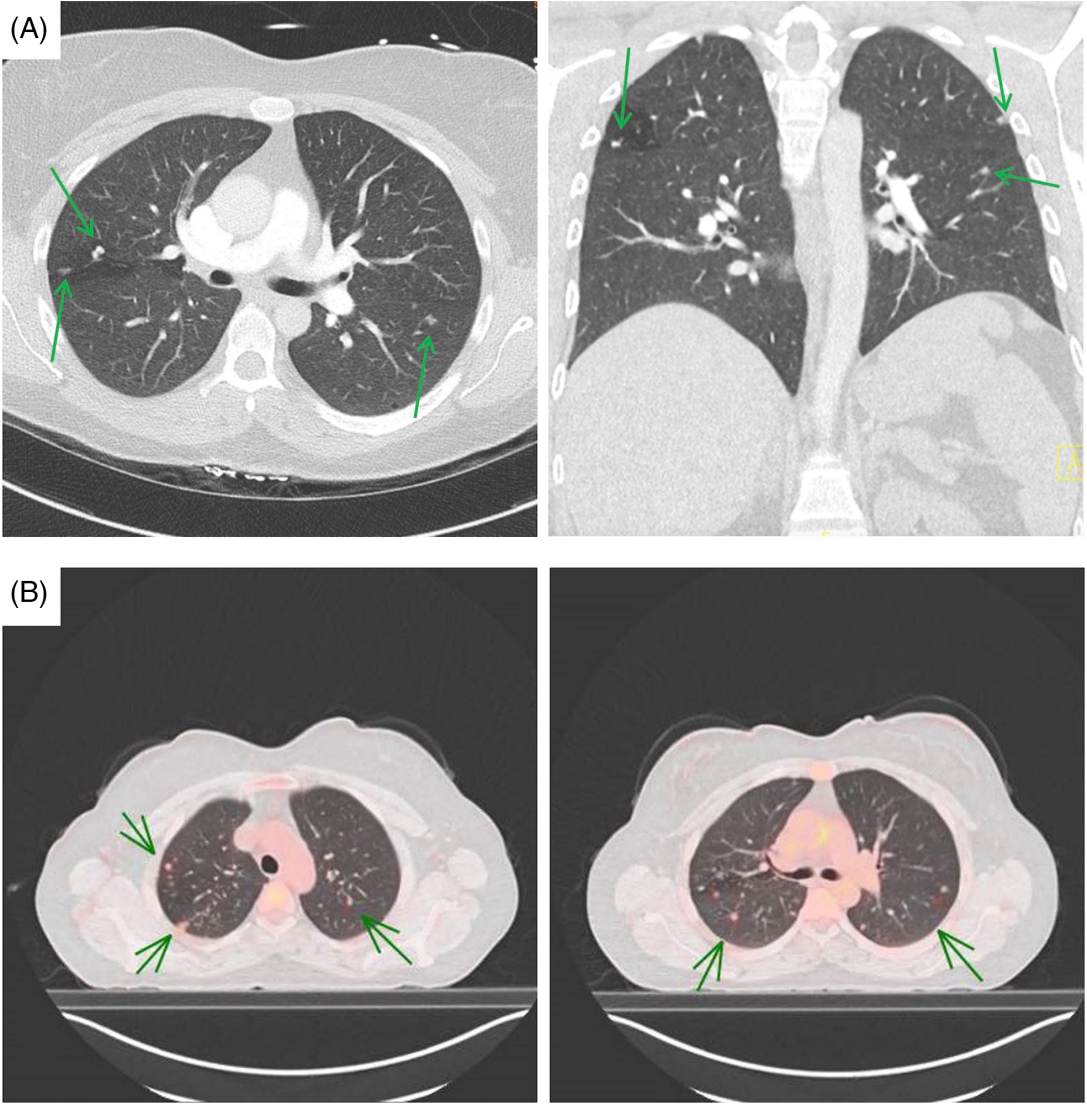
(A) Transverse and coronal slices of initial computed tomography pulmonary angiogram showing multiple bilateral sub‐centimetre nodules in centrilobular distribution. (B) Fused FDG‐PET showing FDG‐avid pulmonary lesions (representative SUV‐max 2.1 subpleural nodule posterior right upper lobe).

The initial CT pulmonary angiogram (Figure 1A) showed multiple bilateral lung nodules, up to 5 mm in diameter and with a mid to upper zone predominance. There was a right hilar lymph node measuring 12 mm and several other prominent bilateral hilar nodes measuring less than 10 mm. A follow‐up CT scan after 6 weeks showed minimally changed pulmonary nodules with no evidence of pathological lymphadenopathy. A subsequent CT scan ruled out any mass lesions in the neck, abdomen and pelvis.

An FDG‐PET scan (Figure 1B) revealed bilateral, sub‐centimetre, FDG‐avid pulmonary lesions although there were no enlarged nor avid lymph nodes. There was no dominant activity to guide sampling. Notably, there was a small focus of mildly FDG‐avid mural thickening in the ascending colon, however a subsequent colonoscopy showed normal colonic mucosa.

Spirometry results indicated normal lung function with an FEV1 3.28 L (101%). Additionally, the diffusion capacity for carbon monoxide was within normal limits.

The differential diagnoses were broad and included inflammatory, granulomatous, and malignant nodules. Due to the size of the pulmonary lesions, which rendered them too small to safely and reliably biopsy via bronchoscopy or CT guidance, she was referred to the cardiothoracic surgeons for a diagnostic surgical biopsy via wedge resection.

**FIGURE 2:**
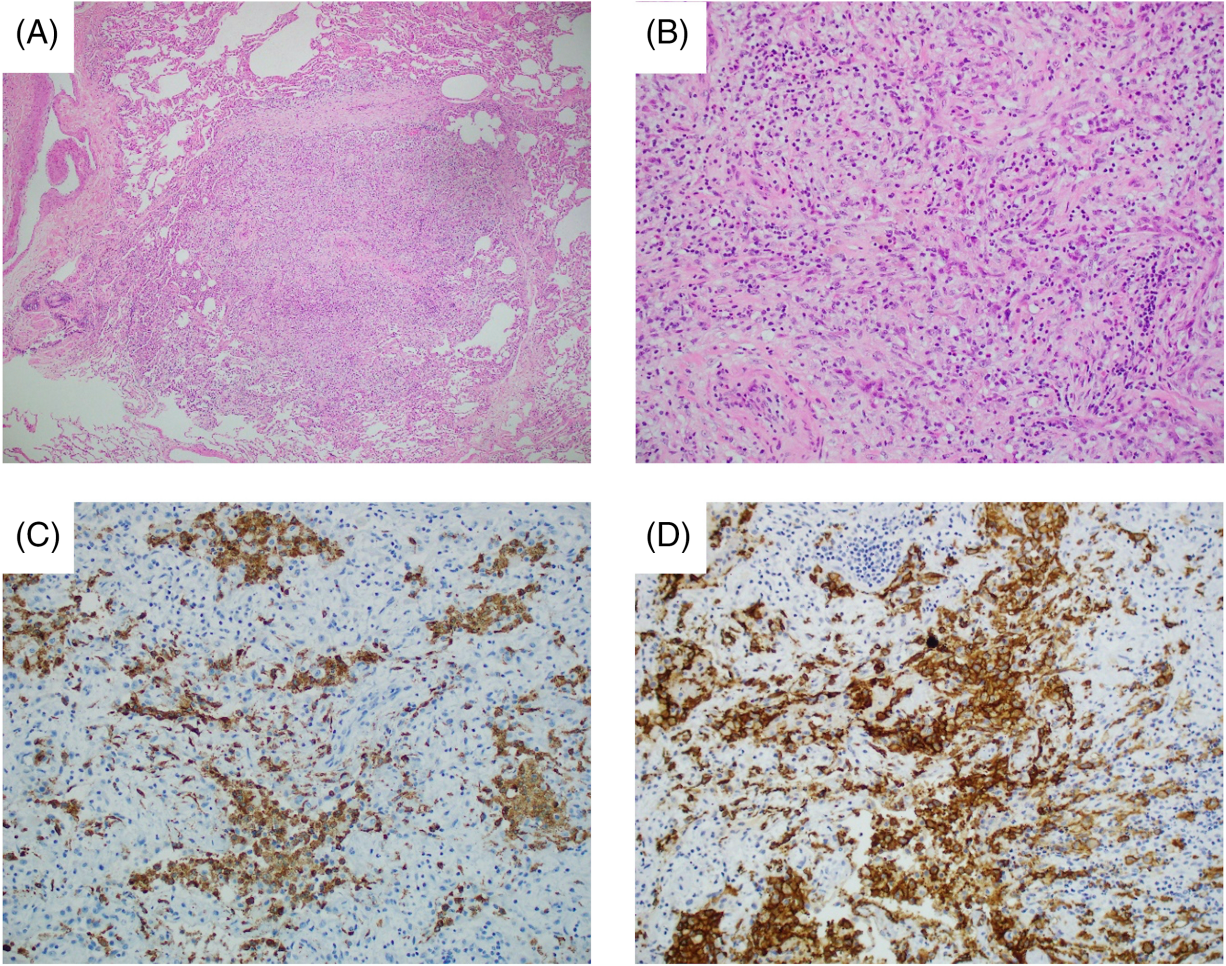
(A) Haematoxylin and Eosin stain ×4 objective magnification showing nodule with surrounding normal alveolar architecture. (B) Haematoxylin and Eosin stain ×20 objective magnification showing occasional eosinophils and numerous Langerhans cells with granular, mildly eosinophilic and indistinct cytoplasm with indented nuclear membranes. (C) Langerin ×20 objective magnification positive staining of Langerhans cells. (D) CD1a stain ×20 objective magnification positive staining of Langerhans cells.

The specimen sections revealed occasional variably sized aggregates of inflammatory cells consisting of a mixed inflammatory infiltrate with prominent numbers of histiocytes and eosinophils. Immunohistochemistry was positive for CD1a and langerin in the histiocytes, supporting a diagnosis of Langerhans cell histiocytosis (Figure 2). BRAF V600E was negative.

**FIGURE 3:**
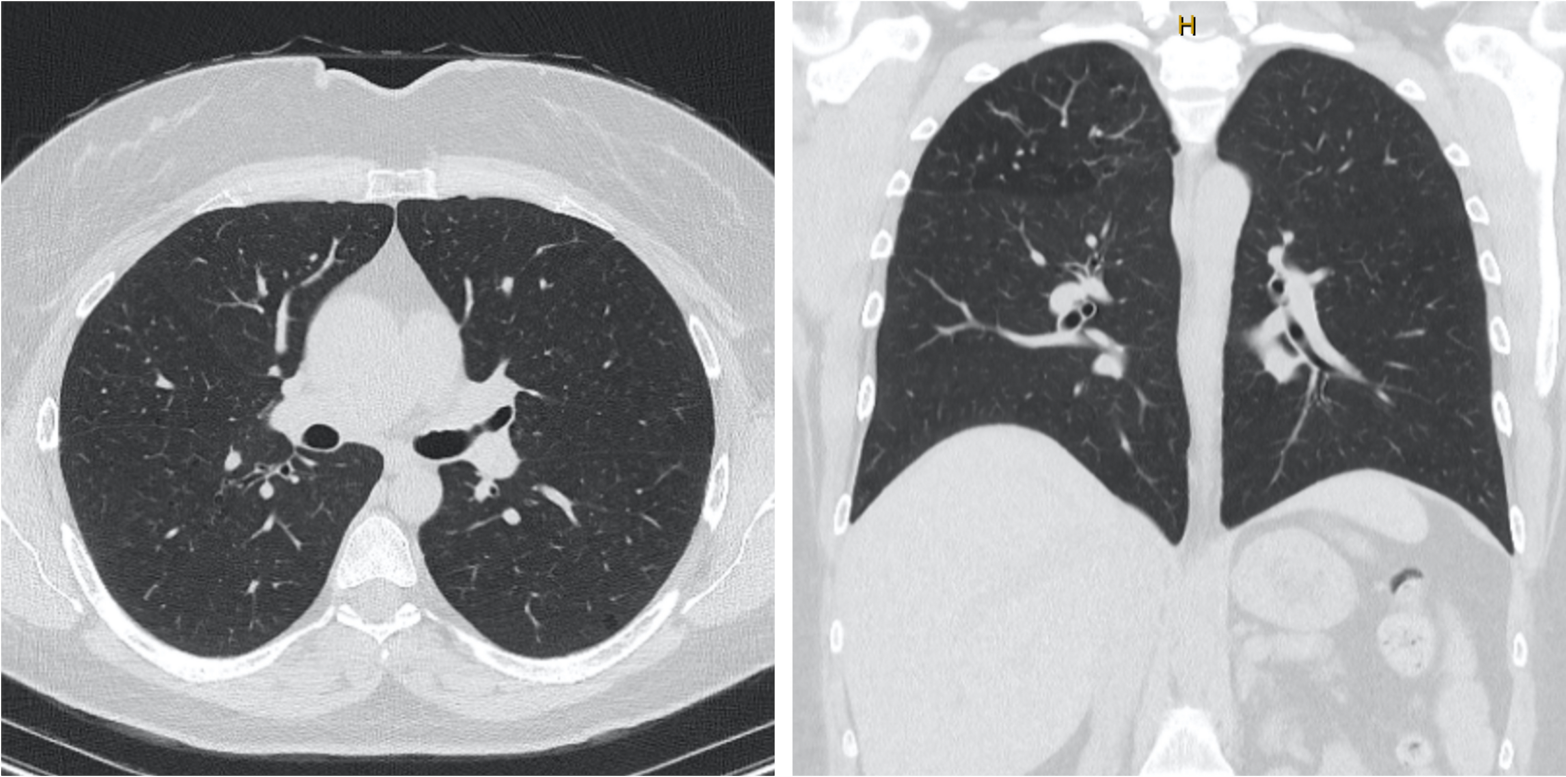
Transverse and coronal slices of repeat computed tomography chest 6 months after diagnosis, showing improvement in the number of pulmonary nodules.

She was counselled on smoking cessation, and after reducing her consumption to less than five cigarettes per day over the following 6 months, a repeat CT chest (Figure 3) showed a reduction in the number of pulmonary nodules although some persisted. Pulmonary function tests remained normal.

## DISCUSSION

Pulmonary Langerhans cell histiocytosis (PLCH) is a rare condition, distinct from other forms of Langerhans cell histiocytosis by clinical and epidemiological factors, although sharing pathologic features. PLCH occurs almost exclusively in smokers and is most prevalent in young adults.^3^, ^5^ Radiologically, it is a heterogenous condition, probably owing to its low burden of clinical symptoms and therefore a variable time period to diagnosis. Serial CT scans in individuals have shown radiological progression from nodules to cavitated nodules, thick‐walled cysts, and finally thin‐walled cysts, and often lesions at multiple stages are seen at one time point.^3^ The pathophysiology behind this progression is largely unknown.^5^ Langerhans' cells (LCs), from the dendritic cell lineage, are predominantly an antigen presenting cell that are found exclusively in the epidermis and mucosal epithelium including the bronchial tree.^4^ Sparce LCs are found in the alveoli under normal conditions. Increased numbers of LCs are seen in smokers, pulmonary inflammation, and some lung cancers.^3^ The progression of granulomas to cyst formation is likely a combination of local inflammatory factors and mediators.^6^ Smoking likely plays a multifactorial role. Osteopontin, a chemoattractant for inflammatory cells, is increased by nicotine and found in higher concentrations in PLCH. Metalloproteases are thought to play a role in tissue destruction in smoking related lung diseases, and may contribute to cavitation in PLCH.^6^ In PLCH, it remains unclear whether the LCH granulomas result predominantly from a neoplastic process involving monoclonal proliferation, or a reactive process with polyclonal expansion, as both have been reported.^7^ The recent identification of BRAF V600E mutation and other MAPK pathway abnormalities in some cases supports monoclonal proliferation in at least a subset of cases.^4^, ^5^

Some cases of PLCH with typical radiological features and little to no symptoms can be managed presumptively with smoking cessation,^3^, ^5^ and the wide availability of high‐resolution computed tomography (HRCT) enables this more frequently. In other cases, the diagnosis can be supported by bronchoalveolar lavage showing non‐specific macrophage predominant cell count, or more specifically >5% CD‐1a positive cells, although this lacks sensitivity.^1^, ^5^, ^6^ Transbronchial biopsy has a limited role in diagnosis, and cryobiopsy is under further investigation.^1^

The radiological features reported in our case, highlights early stages of the pathological progression of PLCH granulomas evidenced by radiological findings of diffuse nodules without cavitation nor formation of cysts, and created diagnostic ambiguity, mimicking multiple more prevalent aetiologies. The case presented here, characterized by typical epidemiological features, concerning clinical features including weight loss, and atypical radiological features, highlights the pivotal role for surgical biopsy to confirm a diagnosis and guide ongoing management, despite advancements in other diagnostic techniques.

## AUTHOR CONTRIBUTIONS

All authors contributed to and reviewed the manuscript.

## CONFLICT OF INTEREST STATEMENT

None declared.

## ETHICS STATEMENT

The authors declare that appropriate written informed consent was obtained for the publication of this manuscript and accompanying images.

## Data Availability

The data that support the findings of this study are available on reasonable request from the corresponding author. The data are not publicly available due to privacy or ethical restrictions.
